# Seeking support for an eating disorder: a qualitative analysis of the university student experience—accessibility of support for students

**DOI:** 10.1186/s40337-022-00562-5

**Published:** 2022-03-07

**Authors:** Nicola C. Byrom, Rachel Batchelor, Harriet Warner, Annie Stevenson

**Affiliations:** 1 Department of Psychology, Institute of Psychiatry, Psychology and Neurosciences, King’s College London, London, UK; 2 Department of Psychology, Royal Holloway, University of London, London, UK

**Keywords:** Student mental health, Eating disorders, Help-seeking, Treatment accessibility, Service-user experience

## Abstract

**Background:**

While there is increased concern around mental health problems in universities, the experience of students with eating disorders (EDs) has received less attention. This is problematic as the detrimental consequences of a lack of adequate support are profound.

**Methods:**

A qualitative study was adopted to investigate students’ perspectives of the availability, accessibility and suitability of support services. One hundred university students with experience of EDs completed an online survey. A further 18 students completed semi-structured interviews. Descriptive data are reported alongside analysis of qualitative data.

**Results:**

Three overarching themes were identified; awareness of support, confidence in asking for help and early experiences with services. Most students were aware of support through their GP and university counselling services. Few identified ED-specific sources of support. Barriers to help-seeking included self-stigma and gaps in service availability. Early experiences with services were often negative, problems included; difficulty accessing services, a lack of ED specific support and continuity of care between home and university.

**Conclusions:**

There is substantive progress to be made in ensuring that students with EDs receive the support they need to thrive at university. While it is important that good treatments exist, the pathway to accessing these treatments equally important. As universities increasingly recognise the need for action around student mental health attention must also be directed towards EDs and the provision of specific services.

## Introduction

There is a pervasive sense of crisis around student mental health. In the UK, university mental health services are reporting increases in demand [1]. Substantive efforts are being directed towards addressing student mental health [2, Universities UK, 3, 4]. Much of the attention currently falls on anxiety and depression.

However, eating disorders (EDs) are common among university students, with prevalence estimates of around 13.5% among women [5–7]. This is unsurprising as the peak age of onset for anorexia nervosa and bulimia nervosa is in the mid to late teenage years [8] and starting university, can be a stressful transition, leading to an increased need for the provision of specialised support [9, 10].

While EDs can be extremely disruptive to university study [11, 12], going to university can be a positive for recovery [5]. However, recovery is significantly influenced by early intervention [13, 14]. Longer durations of illness are associated with poorer outcomes [15, 16] and mental health professionals have concerns about the time delays that students experience in accessing specialist services [13, 17].

The chronic course of EDs makes continuity and integration of care a necessity. This is challenging to achieve given the transient and time-limited nature of student life [18]. There is a treatment gap for university student mental health, with an estimate that only one in five students receives minimally adequate treatment [19]. These gaps are not unique to the student experience and long waiting times are common even for severe EDs [20, 21]. However, students face additional challenges introducing further delays. For example, with both a home and term-time address many students need to be able to access treatment across two geographical locations [9].

While availability of services is a barrier to early intervention [22], encouraging individuals to engage with treatment is also difficult. Stigma and shame act as significant help-seeking barriers [23]. Young adults have a longer duration of untreated illness than adolescents below 18 [24]. New independence from families may allow for a longer period where people have symptoms but don’t recognise these as a problem or feel ready to seek help [13, 25].

When students secure access to support, early interactions can either confirm or disconfirm attitudes towards seeking support [26]. A lack of empathy and training of primary care professionals can adversely impact treatment effectiveness [20] and further reduce help-seeking. It is vital that early interactions with services are positive [27].

In the UK, there are two pathways through which students can access support; through their university counselling service or the National Health Service (NHS). Similar dual pathways occur in other countries, including the USA and Australia. University services commonly provide brief low-level interventions and are not usually designed to support students with EDs [17]. In the UK, students may also seek support via university Disability Advisory Services, with some mental health advisors acting as care coordinators [28]. There are options for students to access a mentor through the Government’s Disabled Students Allowance [29].

With increasing attention on mental health among students, we sought to better understand the experience of students with EDs. We focused on students’ experiences and perspectives, looking at the availability, accessibility and perceived suitability of services. We sought to understand, from the student perspective, whether appropriate services are available and what the student experience is of accessing these services.

## Methods

A qualitative study was employed. Students could participate in an online survey or a semi-structured interview, depending on their preference. The combination of interviews and survey data brings together rich in-depth understand of the issues faced by a smaller number of students, with broader validation of the relevance of concepts among a larger sample. The study was designed in consultation with the student mental health charity, Student Minds and ED charity, First Steps. Co-production has been central to our approach, with the study lead (N.B.) having recovered from an ED. Students (R.B., A.S. and H.W.), some of whom with personal experience of an ED, were involved in all stages of the project. Co-production helps ensure data is collected sensitively and the findings are true reflection of the stakeholder experience [30, 31]. The project was reviewed and approved by the lead institution’s ethics board, through a ‘*high risk’* review process, providing additional scrutiny; reference approval number HR-17/18-6660.

### Participants

Following an information power approach to sample size for qualitative research (Morse, 2000), a relatively small sample size should suffice, as the scope of the study is narrow, the topic of focus is concrete, and in-depth 1-2-1 interviews with interviewers who have a strong understanding of the topic of discussion should facilitate a robust data quality. [32, 33] Students were recruited from across the UK through social media, university recruitment circulars and the mental health charities, Student Minds and First Steps. Participants accessed the study via a webpage hosted by Student Minds, inviting students to help us understand the existing support provision for students with EDs. Inclusion criteria were being over the age of 18 and a current or recent university student. In total, 100 students completed the online survey. A further 18 took part in an extensive, in-depth online interview. Table 1 summarises demographics. Participants were primarily White British females aged 18–24 years old with a self-reported diagnosis of Anorexia Nervosa. A substantive number of survey respondents identified as a sexual minority, primarily bisexual (n = 23). Most respondents had an ED before they moved to university; the most common age of onset was between 13 and 16 years old. Students were primarily undergraduates, representing all years of study. Some respondents were completing a master’s degree (n = 16) or postgraduate research (n = 7).

**Table 1:** Participant demographics

	Survey (N = 100)	Interview (N = 18)	Total (N = 118)
Aged (18–24 years)	85	12	97 (82%)
Ethnicity (White British)	86	16	102 (86%)
Gender (Female)	92	17	109 (92%)
Gender (non-binary)	3	0	3 (2%)
Heterosexual	62	0	0
International student	17	2	19 (16%)
Diagnosis			
Anorexia Nervosa	57	14	71 (60%)
Bulimia Nervosa	16	2	18 (15%)
Binge eating disorder	7	0	7 (6%)
Eating disorder not otherwise specified	20	2	22 (19%)

### Materials

#### Online survey

Respondents were asked whether they thought there were sufficient, appropriate and accessible professional support services for students with EDs and whether they had been able to access support. In a free-text box, survey respondents were asked to list the support that they thought was available to university students experiencing eating difficulties, whether they had considered accessing support from these services and, if not, what their thoughts were around not accessing support from these services. Respondents were then presented with a list of possible sources of support and asked to identify whether they had heard about the service, tried to access support from the service and how helpful they found the service.

Respondents were asked how easy they had found the process of accessing support, given the opportunity to explain more about their process of accessing support and asked what could be done to improve the process of accessing support. Respondents were asked about their experience of the support provided by services and encouraged to identify the most and least helpful aspect of the support provided. Respondents were asked what changes should be made to improve ED service provision.

The General Help-seeking questionnaire (GHSQ) identifies help-seeking intention in young adults and has satisfactory reliability (α = 0.70) and validity while retaining the flexibility to assess intentions across a range of contexts [34]. We used the GHSQ to ask about help-seeking in relation to personal or emotional problems, adapting the scale to include additional support available in universities; (1) the University wellbeing / counselling / mental health team; (2) the University Wellbeing Coaches; (3) a member of the academic teaching team (e.g., a teaching assistant, lecturer or personal tutor). Respondents indicated how likely they are to seek help from a range of individuals, rating their likelihood of help-seeking on a 7-point scale ranging from extremely unlikely through to extremely likely.

#### Interview

The interviews followed a semi-structured topic guide (see Table 2). Interviews aimed to build a full picture of the services that students had accessed, how they accessed the service and whether they had found the service helpful. If applicable, interviewers sought to understand why students had not accessed a common source of support. Students were asked about their experience of moving to university and the impact this had on the support they received. The interviews sought to understand the students’ perspectives on the support provided by the GP, university counselling services, specialist EDs services, academics and university mental health advisors.

**Table 2:** Topic guide

Discussion topic	Follow up prompts
Are you aware of any support available specifically for young adults with eating disorders?	Have you considered accessing any of this support?
What kinds of services have you sought out at university?	How did you access this service? What has your experience been of the process of accessing this support? What were the positive aspects of this service? How could the service have been improved?
What was your experience of moving to university?	Did you have support in place for the Eating Disorder before you moved to university? How did you find the transition to university? Did the services you were using before university help you make the transition to university? When you started at university did you do anything to try to reach out for support?
Have you considered accessing support from the following: GP; University counselling service; Specialist eating disorder service; Personal tutor/academic support; University mental health advisor/disability service	How did you find the experience of seeking support? Did you find this service useful?
In an ideal world, what would have happened, in terms of Eating Disorder support, when you moved to university?	

### Procedure

Students choose to complete the online survey anonymously or request to take part in an interview. Researchers (A.S. or H.W.) screened potential interviewees for inclusion criteria and provided an information sheet and a consent form. A semi-structured interview was used, consisting of open-ended questions, encouraging elaboration. The interview, approximately one hour long, was participant-led; participants were free to discuss, expand on points or change the direction of the conversation. When the conversation came to a natural end, researchers prompted participants by referring to the topic guide. Techniques from motivational interviewing, including non-verbal prompts, were used to encourage participants to elaborate their ideas [35]. Following the interview, participants were debriefed and provided with information on accessing support. Interviews were transcribed.

### Data analysis

Qualitative analysis aimed to identify student perspectives on and experience of service availability, acceptability and suitability. To provide a concise description of perspectives from many participants, a semantic approach was followed; data was taken at face value, with analysis summarising the points and ideas made by respondents.

Thematic analysis was adopted for the interview data (Braun & Clarke, 2006), to provide an opportunity to identify the predominant issues students found important. A reflexive approach was followed, with consideration of how prior knowledge, personal experience and opinions may influence interpretation. Personal experiences of the researchers will have had an influence on the analysis. The team combined lived experience of EDs, including experience accessing support from universities and the NHS. The team had current experience of studying at university and in-depth knowledge of university mental health policies and procedures. As such, the team will have been particularly sensitive to the challenges described by respondents. However, the researchers had limited experience with service design and provision, and thus may have lacked sensitivity in identifying reasons for treatment decisions.

To minimise biases bought by any individual researcher, analysis was completed as a team. Interview transcripts were read and then coded by two raters independently (A.S. and H.W.). Codes were discussed with a third researcher (N.B.) to explore differences in perspectives and agree a final set of codes. Working together similar codes were clustered into themes. Codes that were tangential to the research question and occurred infrequently were discarded. Emerging themes were reviewed, and a holistic approach was taken to ensure that all facets of the data was accounted for. Each theme was named and defined.

Content analysis was adopted for survey responses, given the large number of responses and the need to find responses with the highest relevance. Two independent raters (R.B. and N.B.) read all responses and identified categories of response. Finalised categories were discussed and agreed and the raters worked together to cluster categories with the aim of increasing the coherence of the summary reported. Some participants provided a few words which fit into a specific category, whilst other participants provided several sentences covering multiple categories. Therefore, there was some overlap in participants across categories.

With a full summary of themes from both the interviews and survey, researchers worked together to integrate findings into a single description of the data. To maximise the generalisability of the findings reported, the broad thematic structure from the survey was followed, as this was representative of the experience of a larger number of students. Data from the interviews added depth and enhanced our understanding of the themes identified in the survey. Descriptive analysis of quantitative data was integrated into description of the themes.

## Results

**Fig. 1:**
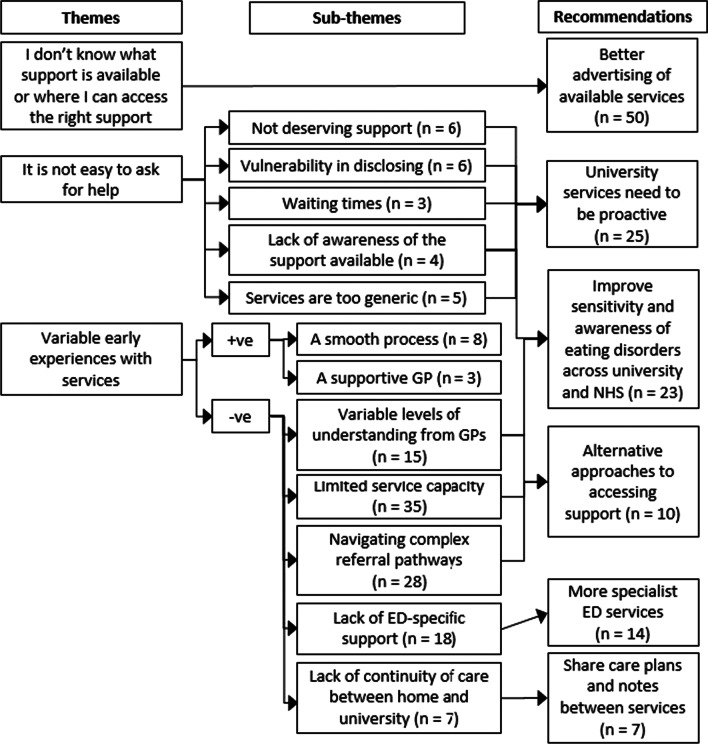
Thematic map, showing themes, subthemes and associated student recommendations

The perspectives and experiences of students fit into three areas, outlined in Figure 1 and elaborated below. These should be viewed in the wider context of respondents describing university as a stressful environment. Academic stressors included frequent deadlines and exams. Social stressors were also described, including alcohol, moving away from home for the first time and living with new people.

### I do not know what support is available or where I can access the right support

Survey respondents were asked to list the support options they were aware of (see Table 3). Students were further asked whether they were aware of or had accessed support from the services listed in Table 3. In contrast to the initial free-text response, most students were aware of common support services. Interviewees demonstrated a similar awareness of services.

**Table 3:** Students identification of support services available

Type of service	Service	Mentions (n)
NHS and University services	GP	36
	University wellbeing services	33
	University counselling service	28
	NHS services	14
	Personal tutors and academic staff	14
	Open door/drop-in sessions with wellbeing advisor	3
	Support available through the Disabled Student’s Allowance	2
	Departmental welfare officer	2
	University psychiatrist	1
	Occupational health	1
	Other non-specific college/university support	3
Charities	Beat	11
	First Steps	10
	Nightline	5
	Student Minds	2
	Mind	2
Other sources of support	Peer and group support	22
	Online resources	6
	Talking therapies	5
	Helplines	5
	Chaplaincy/faith leaders	3
	Private services	2
	Friends	1

However, seven interviewees reported that they would have benefited from better advertising of available services, how to access the services and how these services might be able to help. Many interviewees had only found support through extensive searches. Some participants (interviewees and survey respondents) were still unaware of any suitable services.

“I don’t think I have ever even seen anything to do with eating disorders since I have been at uni until I got the email about this research. I haven’t seen a single poster, not a single anything to do with it.” – Male, 18 – 20, undergraduate (interview)

Students’ suggestions included advertising in a range of places, having more information available about different services and greater signposting to ED services within university; “*Make specific information widespread about any available support.*”

### It is not easy to ask for help

In the GHSQ, students were most likely to identify intending to seek help from a mental health professional, a partner, a friend or a GP. Students were less likely to consider seeking help from a university mental health team or a parent. Students identified that they were unlikely to seek support from an academic, a phoneline, another family member, a university wellbeing coach or a religious minister. Table 4 shows actual help seeking behaviour, confirming that students are most likely to seek support from the GP or university counselling service.

**Table 4:** Student reports of awareness of services, attempts to access services and reflections on the helpfulness of services

	Aware of service	Attempted to access service	Found service helpful/very helpful (as a percentage of those attempting to access the service)
GP	97	58	28 (48%)
Private therapist	95	32	17 (53%)
Helpline	91	15	8 (53%)
University counselling service	86	43	17 (40%)
Advice from a dietician/nutritionist	84	30	15 (50%)
Medication	81	33	15 (45%)
NHS outpatient	74	29	15 (52%)
NHS inpatient	71	12	4 (33%)
Online self-help	74	24	9 (38%)
Peer support programme	64	9	6 (67%)

Just over a third of respondents (35%) identified in the GHSQ that they would not seek help from anyone. Similarly, 31 respondents had not considered accessing support from any of the services listed in Table 4. Of these, two thirds explained why they had not considered accessing support (see Figure 1). Participants feeling undeserving of support reported feeling not ‘ill’ enough to receive help, *“[I] never thought it was bad enough to get help*,*”* not fitting the criteria for services, and not wanting to waste anybody's time, *“I wouldn’t want to waste anybody’s time.”* Vulnerability of disclosing included personal and social concerns. Personal concerns were losing self-control and admitting that they had a disorder. Social concerns related to the reactions of others such as being judged or misunderstood and lacking trust in others. This theme was echoed in the student interviews, where 11 interviewees stated that they had found it difficult to seek support because of shame, stigma and a lack of motivation.

“It is really difficult to disclose one's struggle with such mental disorder to someone from the "outside" world as it makes one feel out of control (self-control) and more vulnerable. Although one might feel that it is the right time to seek help, it is at the same time extremely difficult to admit to oneself that he/she is suffering from such disorder. Equally, there is a fear of being judged or misunderstood so often people would not opt for such services.” - Female, 21-24, undergraduate (survey)

Addressing the challenges of help-seeking, respondents suggested that steps should be taken to improve ED understanding and awareness across universities. Participants described a need for educating students and university staff about the different types of EDs, early signs of EDs, and how to help to practically support students with an ED through university (e.g. with meals, course adjustments). Participants hoped such awareness would reduce social stigma surrounding EDs and ensure students with EDs are listened to and met with compassion.

“Providing information about the signs of a developing ED and how to recognise it in yourself/friends.” - Female, 18-20, undergraduate (survey)

Respondents identified a need for university services to be proactive in addressing help-seeking difficulties, suggesting universities should actively make students aware of services, encourage students to seek support, offer early intervention and employ student wide screening to identify those at risk. Some students describe receiving an offer of support from the university when they arrived. This was often dependent on effective communication from the services supporting the student prior to moving to the university. However, 28% of interviewees felt that no-one within their university noticed their struggles and no one would intervene.

‘’I think there should be regular screenings for most students (short surveys or assessments sent out by personal tutors) so that students who don't seek out help can at least be flagged.” - Female, 18-20, undergraduate (survey)

### Variable early experiences with services

As shown in Table 4, for most services, of the students accessing these, only half found them to be helpful. Of the 63 survey respondents who had considered accessing support, 53 summarised their experiences of this process, outlined in Figure 1. Four participants shared only positive experiences, 47 shared only negative experiences and 10 shared a mixture of the two. The responses provided by two participants did not fit within these categories, due to being too vague or not reporting their own experience. In interviews, 13 students identified examples of good care and 14 identified examples of poor care. Within negative experiences of accessing ED support, the categories listed in Figure 1 were highly inter-linked and inter-dependent. We provide further detail on these categories below.

#### Variable levels of understanding from GPs

When discussing experience with GPs, most respondents described challenges (see Figure 1). This was echoed in survey data where only 48% identified their GP as helpful (see Table 4). Difficulties seeking help from the GP included GPs being perceived to be judgemental, unempathetic, not understand EDs and not respond to requests for support; *“I didn’t like the GPs, they were really insensitive to mental health”.* Where interviewees described positive experiences, GPs were described as good listeners, understanding and empathetic. Interviewees identified a need for more sensitivity and awareness across university and NHS services. Participants perceived that a good understanding of EDs would be a benefit to the process of seeking support as well their overall university experience.

“I think it so depends on the GP that you see, and their understanding. If you get one that’s really nice and actually kind of understands what they’re on about, it can make or break your experience.” - Female, 21 – 24, undergraduate (interview)

#### Limited service capacity

Many participants identified a need for all services to be accessible, where students could attend appointments easily, without long travel times or clashes with their academic commitments, *“I had an appointment and it was far away from campus…it took me an hour and a half.”* Limited service capacity reduces accessibility. Participants reported long waiting lists, short appointments, limited treatment durations, and insufficient staff numbers.

“Waiting lists for university counselling is too long and the sessions are too short to build rapport with your counsellor or make any changes.” - Female, 18-20, undergraduate (survey)

Alternative approaches to increasing access to services were suggested, including befrienders, peer and group support (*n* = 5), online support (*n* = 3), drop-ins (*n* = 3), mentoring between staff and students (*n* = 1) and practical support with meal plans (*n* = 1).

“More online and befriending support. Peer support is great if you're lucky to be able to access it at your Uni.” - Male, 21-24, undergraduate (survey)

#### Navigating complex referral pathways

Participants identified challenges relating to treatment criteria, with referral thresholds being too high, or the criteria too limited. Interviewees described being told that their BMI was either ‘too high’ or ‘too low’ to qualify for treatment.

“As someone who struggled with bulimia, I am a normal weight. Consistently been told I can't access specialist services as I am not underweight.” - Female, 18-20, undergraduate (survey)

“When my [ED] issues first started I couldn't access NHS help due to the guidelines stating I needed a BMI [below] 17.0 (despite displaying every other symptom) which was taken as encouragement to continue.” - Female, 21-24, undergraduate (survey)

Comorbidities further limited access to services provided by the NHS, university mental health teams and charities. Complex referral pathways can lead to students being passed between services. Ten participants reported being passed between services whilst trying to access support. Participants described often having to go through several steps and teams and repeat their situation over and over, *“lots of repetitiveness and having to explain yourself to lots of different people, a draining process.”*

In contrast, some respondents described a smooth process of accessing support included this being easy, efficient and flexible.

“Surprisingly easy and flexible, appointments have been quick and easy to set up, and have been organised remotely when I've needed this option because of my disabilities.” - Queer, 25-34, master’s student (survey)

In terms of a smooth process, four participants recognised that it was easier for them to access support due to the severity of their difficulties and being considered high risk, *“[The] only reason at [blank] it was quick was because I was at a high suicide risk.”* An additional two participants specified that they perceived themselves as ‘lucky’ for managing to access support *“I was ‘lucky’ in that my BMI at the time of seeing a GP made access to support easy.”*

#### Lack of eating disorder specific support

Most interviewees felt that given the complexity of EDs, support needed to be provided by trained staff through specialist services. Unfortunately, some participants noted that the treatment they had received from university and NHS services was not adequate to provide appropriate care; “*there was a counselling service, but they didn’t have the relevant expertise or knowledge*”.

In some cases, this prevented students from returning to services for support. Students suggested that there is a need for more specialised ED services. This included the provision of evidenced-based treatments for EDs, designated services, and training staff to have a better understanding of comorbid conditions and how EDs can affect all aspects of life.

“Make sure there are more specialised support groups for students with eating disorders and not just general 'low mood'. Make sure staff are better trained about EDs as many lack the training to deal with issues like this.” - Female, 18-20, undergraduate (survey)

#### Lack of continuity of care between home and university

Participants identified a lack of continuity of care between home and universities and experienced long waiting times to access NHS services and university mental health services. Participants were usually managing without any support during this time. Participants described difficult transitions, being unable to register with two GPs or receive support at certain times due to living between two places. In moving to university students experienced dramatic changes in the intensity of support.

“Difficult to access support when ‘home’ and university services do not connect. A student will be in two places, each for roughly half the year. This gives an inconsistent level of care. I have found myself moved from waiting list to waiting list” - Female, 21-24, undergraduate (survey)

“I went from all of that support to nothing… it was really tough. I took some back steps during that time.” – Female 18 - 20, undergraduate (interview)

The challenge of gaps in care was further compounded for many by poor communication between services. While 28% of interviewees specifically noted examples of good communication which were a real benefit to them, 61% had experienced poor communication. Participants identified a need for home and university collaborative care.

“No one ever spoke, no one ever communicated, and I think that lack of communication was just really frustrating because I felt I repeated myself all the time” - Female, 25 - 34, recent graduate (interview)

Looking for effective communication, students would like care plans and notes to be shared between services. Any steps to reduce the need to keep ‘sharing their story’ were appreciated. Good communication takes the pressure off the individual to manage their own care. Students would like to be able to start the referral process before moving to university.

“Being able to be registered with services in two places (e.g. parents’ home and university) or flexibility in stopping treatment for a few weeks (e.g. over university holidays). Being able to get a referral started before moving somewhere. I had to wait until I moved to a new city and had an address to be able to register with a GP and get the referral process started.” - Female, 21-24, postgraduate research student (interview)

“I think being able to be with two GPs would help so you can stay under your home team. Or communication between NHS area services so transition can be smooth without more assessments.” - Female, 21-24, undergraduate (survey)

#### Consequences

**Fig. 2:**
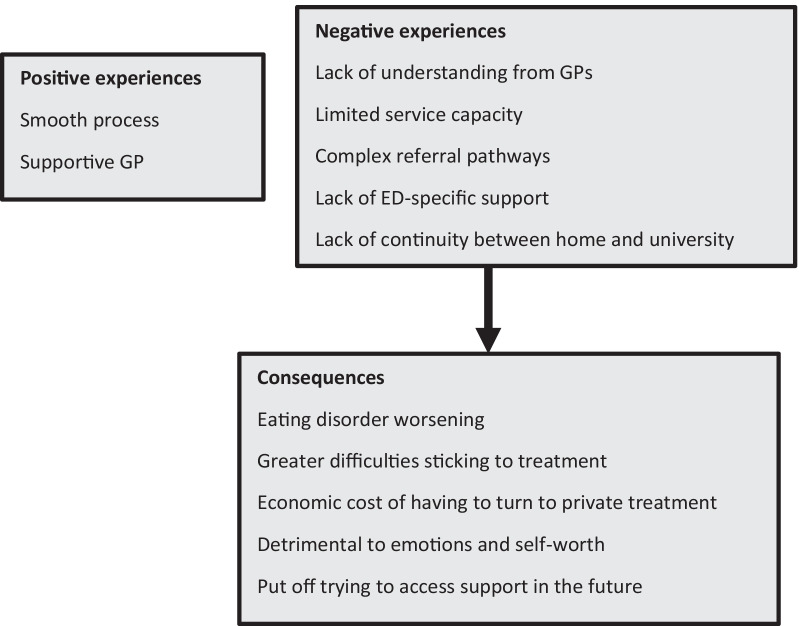
Categories from the content analysis on qualitative responses for experiences of the process of accessing support

Reflecting on these challenges, ten survey participants went on to describe the consequences of their negative experiences with support services, summarised in Figure 2. These included their ED worsening, having greater difficulties sticking to treatment, the economic cost of having to turn to private treatment, a detriment to emotions and self-worth, and being put off trying to access support in the future.

“Very long waiting lists for lots of services, which makes you feel as if you are not important, ultimately making the situation worse.” - Female, 18-20, undergraduate (survey)

“You have to go through lots of steps and are handed down through different people and may find at the end that it’s not the right help for you, which is off putting for the future.” - Female, 18-20, undergraduate (survey)

## Discussion

### Summary of main findings

This study explored students’ perspectives of accessing treatment for an ED at university, integrating qualitative data from in-depth interviews and a large survey. While most students were aware that they could access support through a GP, private practices, helplines and university counselling services, few students mentioned ED specific sources of support, and many seemed unaware of support available through the Disabled Students Allowance or charities. Approximately a third had not considered accessing any support for their ED. Substantive barriers to help-seeking were identified including self-stigma, vulnerability associated with disclosing and gaps in service availability. Respondents’ early experiences with services were often negative. Difficulties accessing support included limited service capacity, complex referral pathways, including strict limitations on referral criteria, and services not providing ED specific support. Students identified a lack of understanding from GPs and a lack of continuity of care between home and university. Students felt that they were not receiving the help that they needed from either university or GP services. This support gap was worsened by a lack of clarity and shared expertise and understanding of the roles between university and health services.

### Finding information about support services

Identifying how and where to seek support was a challenge, with students showing poor understanding about the support available or how this can be accessed. Greater publicity of support services available for students with EDs is needed. This should include an explanation about how the service works, how it can be accessed and what support it can provide. Most students were aware that they could access support through their GP or university counselling services, however a lack of understanding from GPs could be a barrier to accessing treatment. Where specialist ED services are available, options to access these through an initial drop-in appointment may be preferable.

Services publicised must have the training, experience and skills to support students with EDs. Early interactions with services are important for shaping motivation and enthusiasm to engage with treatment [26]. Overwhelmingly students were describing poor early interactions. This was in part because students are reaching out to services, such as university counselling services, that are not designed to support individuals with EDs. Clinicians with specialist expertise in treating EDs have identified similar concerns about the ability of GPs and university professionals to detect and manage students with EDs [36].

### Barriers to accessing treatment

Students reported barriers to accessing treatment including self-stigma, vulnerability associated with disclosing an ED and an inability to identify a source of specific support. Such barriers are not unique to eating disorders and have been reported amongst university students with a range of mental health difficulties [37], along with concerns that disclosure may impact their course progression [38]. Disclosure and access barriers are also not unique to students. Self-stigma is a substantive challenge, with individuals not feeling they are deserving of support, not wanting to lose control or admit they have a problem, and being worried about being judged by others [39]. While there have been efforts to tackle stigma around mental health, self-stigma still presents a barrier to help-seeking [37, 40]. Self-stigma and ambivalence about accessing support must to be kept in mind when considering the steps that should be taken to improve support for students with EDs [18, 23]. Despite the applicability of such findings to wider populations of mental health conditions and non-university students, the chronicity and poor outcomes of EDs makes timely support crucial [14, 15].

One option is to address disclosure and access barriers is to adopt a more proactive approach. Students would like staff across universities to have a better understanding of EDs, to identify when a student might be struggling and connect them with services. Better training around EDs for university staff would be valuable [36]. All mental health training for university staff should consider EDs and identify what steps can be taken to encourage students to seek support. Previous research in schools identified that teachers did not feel comfortable talking to students about ED, but would welcome practical ideas for how they can support students during recovery [41]. Similar research in universities may help to develop effective training to support academics. It is essential for such an approach to be managed with care, recognising the existing challenges that academics face in maintaining boundaries when supporting students [42, 43]. It may also be appropriate to provide specific training for other university staff, such as Students Union officers and staff in halls of residence and sports centres [44].

### Early experience with services: university provision

Students were accessing support through their university counselling services. This appeared to be a frustrating experience, with students describing long waiting times followed by a lack of satisfactory support. University services are not designed to manage EDs. Arguably it is not a university’s responsibility to provide such specialist support. Often these services are supporting students because there are gaps in care or students are unable to access specialist treatment through the NHS [45]. In these cases, there is a sense that ‘something is better than nothing.’ University services have two non-mutually exclusive options.

First, services could improve staff training around EDs to ensure that students receive support from someone who understands their experience and the support they need [36]. Ensuring that there is a named member of staff within the service who has specific training in EDs could improve the approachability of the service.

Secondly, services need to be clear about their limitations and manage student expectations. It is essential that students are engaged in a discussion that sets expectations and explores what support someone without specialist expertise can provide. Services must ensure that students do not spend time waiting to access a service before being told that the service cannot support them.

### Early experience with services: NHS provision and continuity of care

Ideally students with EDs should be able to access appropriate treatment through health services. There are universal challenges to this, with limited referral criteria acting as a barrier to accessing treatment and long waiting times delaying support [13]. Referral criteria are acting as a barrier to accessing treatment, with students repeatedly mentioning that BMI was a factor in referrals. While these issues are unlikely to be specific to university students, they are affecting students. The majority of eating disorders are not accompanied by very low BMI [46] and it is common for individual with an eating disorder to have comorbidities [47]. However, the presence of comorbidities and the absence of very low weight were identified here as barriers to accessing treatment. The focus on weight in referral criteria may exacerbate unhealthy cognitions, encouraging individuals to define themselves by their weight. The 2019 NHS guidance highlights that decisions on referrals should never be made solely on a person’s BMI and notes that having a comorbid condition should not be a reason for rejecting someone from treatment [48].

Students experience additional challenges accessing treatment due to their dual residency. While efforts have been taken to allow ‘dual registration’ in general practice [49], when students first move to university and then subsequently move between home and university throughout the year, substantive gaps care may arise [9, 17, 36].

Smooth handovers between treatment teams has been identified as imperative to ensure the sustainability of treatment outcomes [50]. A smooth transition when a young person enters university is especially important because this is a major life transition, recognised to be a risk factor for EDs [9]. The NHS England commissioning guide for adults with EDs includes a specific section on support for students, including the need to manage transitions effectively [48]. However, students are experiencing substantive treatment gaps around the transition to university. For most of the students we spoke to, their ED started before they moved to university and many had specialist support in place before they moved to university. Upon moving, these students either experienced a complete break in their care or a decrease in the level of support they were receiving.

Treatment gaps are compounded by a lack of communication between services. Concerns about communication between services are echoed by clinicians. Our findings support calls for the development of specific national guidelines to enhance coordination of care and communication across services as young people transition to university [36]. While links should be made within the NHS, university mental health advisors or disability advisors should play a role in care coordination and take steps to ensure that plans for support are made in advance of a student moving to university [17, 28].

### Early experiences with services: the burden of coordinating care

For several reasons, including self-stigma, self-control and lack of motivation, it can be hard to say, “I think I have an ED; I need help.” However, as reflected in our findings, the current complex referral pathway and siloed treatment services that young people must navigate often demands that they have to say this repeatedly. This repetition may encourage damaging internalisation of an eating disorder narrative, further shaping identity around the eating disorder. Where consented, sharing care plans and notes between services would lessen the burden on students. Development and appropriate sharing of comprehensive care plans is part of the NHS England recommendation [48]. Students, and if appropriate their families, should be involved in a decision-making process and provided with documentation outlining key elements of their medical history and care plans [17]. More students may be encouraged to engage with treatment, if steps are taken to reduce the burden they experience in managing their own care [51].

Where students are known to have an ED, efforts should be taken to ensure adjustments are provided (e.g. flexible attendance to accommodate access to mental health appointments) and their condition is monitored. From the university’s perspective monitoring may be about arranging for more regular personal tutor check-ins when a student has disclosed a mental health problem. However, given concerns about blurred boundaries for the personal tutor role [42, 43], it may be more appropriate for regular support to be provided through a mentor funded by the Disabled Students Allowance. Very few students mentioned this form of support, indicating that mentors may be under-utilised; universities have a responsibility to make information about this type of support widespread. Within the health service, GP practices should ensure that the student has a named GP and are able to see them.

### Limitations

In integrating survey responses from many students with in-depth interviews and recruiting students from across the UK, this study provides a comprehensive review of the student experience. However, despite considerable efforts through recruitment, our study under-represents men and individuals from ethnic minorities. We suspect that these individuals face additional barriers to accessing treatment, with poor recognition of the support men need for EDs [52] and concerns about the cultural competency of support services [53]. Further targeted work is needed to understand the experience of these students.

While this study was open to all students experiencing EDs, we have primarily heard from individuals with Anorexia Nervosa. This is unusual; previous studies into barriers for accessing treatment have tended to under-represent individuals with Anorexia Nervosa [22]. In this study students self-described their ED. Given the tight diagnostic criteria for anorexia nervosa, it is possible that a proportion of those identifying as experiencing Anorexia Nervosa, might be diagnosed by a clinician with a more general description of OSFED (Other Specified Feeding and Eating Disorders). OSFED is estimated to be at least twice as prevalent as Anorexia Nervosa and Bulimia Nervosa has a similar incident rate to Anorexia Nervosa [46] and yet individuals with OSFED and Bulimia Nervosa were under-represented in this study. From the data we have collected we suspect that individuals with Bulimia Nervosa may face additional challenges accessing specialist support, as students identified the use weight as a referral criterion being a barrier to accessing treatment. Further research is needed to understand the experience of students with Bulimia Nervosa. This work needs to specifically target students with this diagnosis to ensure that their experience is heard.

### Conclusions

In 2013 Student Minds published a report into the challenges that students face accessing treatment for EDs [17]. Many of the challenges identified there are echoed here, such as difficulties with navigating siloed treatment services, indicating that there is still substantive progress to be made in ensuring that students with EDs receive the support they need to thrive at university. While it is important that good treatments exist for EDs, the care pathway that surrounds the experience of accessing these treatments is also important. Our findings reiterate the challenge that students experience in acknowledging that they have an eating disorder and reaching out for help. In this context, help-seeking needs to be simple and supported. Our research indicates that for EDs, this care pathway requires substantive attention. This will require renewed efforts from universities and the NHS. Suggested improvements identified by our study include greater publicity of support services, ED training for support and university staff, co-ordination between treatment teams and the involvement of students in decision-making processes surrounding their care. The description of positive experience, including GPs being empathetic and appointments being easy to arrange, highlights the potential to develop good treatment pathways. As universities increasingly recognise the need for action around student mental health [2, 4, 54], it is essential that attention is directed towards severe and chronic mental health problems, including EDs.

## Data Availability

Given the sensitive nature of this project and the in-depth qualitative data collected, we did not seek consent to make data publicly available.

## Abbreviations

ED: Eating disorders
GP: General practitioner
GHSQ: General Help-Seeking Questionnaire
OSFED: Other specified feeding and eating disorders
NHS: National Health Service
